# ICAT promotes colorectal cancer metastasis via binding to JUP and activating the NF‐κB signaling pathway

**DOI:** 10.1002/jcla.24678

**Published:** 2022-08-29

**Authors:** Zihan Wang, Jiancong Hu, Junxiong Chen, Jingdan Zhang, Weiqian Li, Yu Tian, Huanliang Liu, Xiangling Yang

**Affiliations:** ^1^ Department of Clinical Laboratory, The Sixth Affiliated Hospital Sun Yat‐sen University Guangzhou China; ^2^ Guangdong Institute of Gastroenterology Guangzhou Guangdong China; ^3^ Guangdong Provincial Key Laboratory of Colorectal and Pelvic Floor Diseases, The Sixth Affiliated Hospital Sun Yat‐sen University Guangzhou Guangdong China; ^4^ Department of Colorectal Surgery, The Sixth Affiliated Hospital Sun Yat‐sen University Guangzhou Guangdong China

**Keywords:** CRC, ICAT, JUP, metastasis, NF‐κb

## Abstract

**Background:**

The inhibitor of β‐catenin and T‐cell factor (ICAT) is a direct negative regulator of the canonical Wnt signaling pathway, which is an attractive therapeutic target for colorectal cancer (CRC). Accumulating evidence suggests that ICAT interacts with other proteins to exert additional functions, which are not yet fully elucidated.

**Methods:**

The overexpression of ICAT of CRC cells was conducted by lentivirus infection and plasmids transfection and verified by quantitative real‐time reverse transcription‐polymerase chain reaction (real‐time RT‐PCR) and Western blotting. The effect of ICAT on the mobility of CRC cells was assessed by wound healing assay and transwell assay in vitro and lung metastasis in vivo. New candidate ICAT‐interacting proteins were explored and verified using the STRING database, silver staining, co‐immunoprecipitation mass spectrometry analysis (Co‐IP/MS), and immunofluorescence (IF) staining analysis.

**Result:**

Inhibitor of β‐catenin and T‐cell factor overexpression promoted in vitro cell migration and invasion and tumor metastasis in vivo. Co‐IP/MS analysis and STRING database analyses revealed that junction plakoglobin (JUP), a homolog of β‐catenin, was involved in a novel protein interaction with ICAT. Furthermore, JUP downregulation impaired ICAT‐induced migration and invasion of CRC cells. In addition, ICAT overexpression activated the NF‐κB signaling pathway, which led to enhanced CRC cell migration and invasion.

**Conclusion:**

Inhibitor of β‐catenin and T‐cell factor promoted CRC cell migration and invasion by interacting with JUP and the NF‐κB signaling pathway. Thus, ICAT could be considered a protein diagnostic biomarker for predicting the metastatic ability of CRC.

## Graphical abstract

Inhibitor of β‐catenin and T‐cell factor (ICAT) promotes colorectal cancer (CRC) cell migration and invasion by interacting with the junction plakoglobin (JUP) and via the NF‐κB signaling pathway in CRC.

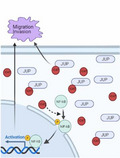

## 1 INTRODUCTION

Colorectal cancer (CRC) is one of the most common malignant cancers worldwide, ranking third in terms of both incidence and mortality.^1^ Recurrence and metastasis are major lethal factors in CRC; thus, it remains critical to assess our current understanding of CRC and develop novel therapeutic strategies and diagnostic biomarkers.^2^

The inhibitor of β‐catenin and T‐cell factor (ICAT) is composed of 81 amino acids, which was initially isolated by utilizing yeast two‐hybrid screening using β‐catenin as a bait protein.^3^ Subsequently, the crystal structure of the β‐catenin/ICAT complex revealed that the inhibitory mechanism of ICAT selectively inhibits β‐catenin/T‐cell factor (TCF) binding in the nucleus, without disrupting the β‐catenin/cadherin complex in the cytoplasm.^4^ In general, ICAT can disrupt the β‐catenin/TCF/DNA complex by inhibiting the binding of β‐catenin and TCF, which significantly reduces the activation of the target genes of the β‐catenin/TCF complex.^5^, ^6^, ^7^, ^8^, ^9^ Thus, ICAT is an attractive target for CRC therapy. However, accumulating evidence indicates that ICAT may have diverse functions in different tumors. For example, ICAT has also been shown to promote the motility and metastasis of melanoma and liver cancer.^10^, ^11^ Another study showed that ICAT inhibited the binding of β‐catenin to E‐cadherin, which promoted tumor metastasis.^12^ This finding suggests that ICAT interacts with other proteins to perform different other functions. Our previous study demonstrated that ICAT is located in the cytoplasm of CRC cells and that the interaction of ICAT with the Wnt signaling pathway regulates the proliferation of CRC cells.^13^ However, the role of ICAT in CRC metastasis and the proteins that interact with ICAT are not fully elucidated.

Junction plakoglobin (JUP), a homolog of β‐catenin, is an important cell–cell junction protein related to adhesion junctions and desmosome composition.^14^ The canonical function of JUP is linking E‐cadherin to the actin cytoskeleton as well as desmocollins and desmogleins to the intermediate filaments of the actin cytoskeleton in desmosomes. Researchers have found that JUP is highly expressed in several malignancies, including breast cancer,^15^ acute myeloid leukemia,^16^ and lung adenocarcinoma.^17^ JUP can serve as a potential prognostic biomarker, and high JUP expression is associated with poor clinical outcomes. However, there have been only a few studies related to JUP in CRC.

In this study, we aimed to investigate the novel roles and interacting partner of ICAT involved in CRC cell migration and invasion in vitro and in vivo.

## 2 MATERIALS AND METHODS

### 2.1 Cell culture

Colorectal cancer cell lines (SW480 and HCT116) were purchased from the American Type Culture Collection (USA). CRC cells were cultured in RPMI 1640 (Gibco) medium containing 100 U/mL penicillin–streptomycin and 10% fetal bovine serum (FBS) at 37°C and 5% CO_2_.

### 2.2 Lentivirus infection and transfection

Stable cell lines overexpressing the protein of interest were developed as previously described.^13^, ^18^ Lentiviruses carrying ICAT cDNA and vector control (VC) packaged by Vigene Biosciences were transfected into the SW480 cell line following the manufacturer's instructions to construct ICAT‐overexpressing cell lines and VC. Stable cell lines were selected after 2 weeks of puromycin (10 μg/ml) selection and were maintained in 1 μg/ml puromycin.

For transfection, cells were cultured in six‐well plates for 24 h. The pLVX‐IRES‐puromycin vector with the ICAT insert tagged with Flag was used to transiently overexpress ICAT. The plasmids were transfected into HCT116 cell lines using Lipofectamine 3000 (Life Technologies). siRNA against JUP and the negative control (50 nM, Tsingke Biotechnology) and siRNA against P65 and the negative control (20 nM, GenePharma) were transfected into HCT116 cells using Lipofectamine® RNAiMAX (Invitrogen). The transfection efficiency was measured by Western blotting and quantitative real‐time reverse transcription‐polymerase chain reaction after 48 h.

### 2.3 Transwell assay

Transwell cell migration and invasion assays were performed as previously described.^19^ Briefly, we used Matrigel (BD Bioscience) to coat transwell chambers to perform the invasion assay, and Matrigel chambers without pre‐coated for the migration assay. When the cells attained logarithmic growth phase, they were digested with trypsin, washed twice with serum‐free medium, and suspended in serum‐free medium for counting. Cells (2 × 10^5^) were suspended in 200 μl of serum‐free medium, and were added to the upper chamber of the transwell chamber, and 700 μl of complete medium was added to the lower chamber to induce cellular migration. After 24 or 36 h of incubation, the transwell chamber was removed, and the cells were fixed in methanol for 10 min and then stained with crystal violet for 10 min. Non‐migratory cells were carefully wiped from the indoor layer of the chamber with a cotton swab, and the number of cells that had migrated to the outdoor layer of the chamber was visualized and counted using a microscope (OLYMPUS CKX41) and ImageJ software.

### 2.4 Wound healing assay

The four‐well culture insert (Ibidi) was placed in the middle of a 12‐well plate. Cells (3 × 10^5^) were suspended in 500 μl of the complete medium. 110 μl of the suspension was added into four reservoirs of the culture insert. After 24 h of incubation, we removed the culture insert and added 1 ml of complete medium into the well. Wound closure was monitored using a microscope (OLYMPUS CKX41) over a period of 24 h and the rate of wound closure was analyzed using ImageJ software.

### 2.5 Western blotting

Cells were lysed in RIPA lysis buffer (Beyotime), separated by SDS‐PAGE, and transferred onto PVDF membranes (Millipore). Membranes were blocked with 5% defatted milk for 1 h and incubated with anti‐ICAT (A7122, 1:2000, ABclonal), anti‐ICAT (ab129011, 1:2000, Abcam), anti‐JUP (381,616, 1:2000, *ZEN‐*Bioscience), anti‐GAPDH (10494‐AP, 1:5000, Proteintech Group), anti‐phospho‐P65 (p‐P65) (AF5881, 1:1000, Beyotime), or anti‐P65 (AF0246, 1:1000, Beyotime) primary antibodies at 4°C for 10 h. The membranes were washed thrice with TBST for 5 min each and incubated with horseradish peroxidase‐conjugated secondary antibodies to rabbit (#820112, 1:2000, EarthOx) or mouse (#620112, 1:2000, EarthOx) for 1 h. The signals were detected using a chemiluminescence system (Bio‐Rad).

### 2.6 Quantitative real‐time reverse transcription‐polymerase chain reaction (real‐time RT‐PCR)

The total RNA was isolated from cancer cells using an RNA‐Quick Purification Kit (ES science Biotech). RNA (1 μg) was reverse‐transcribed into cDNA using the PrimeScript RT Master Mix (Takara Biomedical Technology). Real‐time RT‐PCR was performed using SYBR GREEN Premix Ex Taq II (Takara Biomedical Technology) on an Applied Biosystems QuantStudio 7 Flex (Life Technologies). The results were analyzed using the 2^‐ΔΔCT^ method. The following primers were used:

CTNNBIP1‐F: 5′C‐CTATGCAGGGGTGGTCAACA‐3′,

CTNNBIP1‐ R: 5′‐CTGGAAAACGCCATCACCAC‐3′,

GAPDH‐F: 5′‐GCACCGTCAAGGCTGAGAAC‐3′,

GAPDH‐R: 5′‐TGGTGAAGACGCCAGTGGA‐3′.

### 2.7 Animal experiments

The animal experiments (IACUC‐2020050602) were approved by the Committee on the Ethics of Animal Experiments of the Sixth Affiliated Hospital, Sun Yat‐sen University. Cells were resuspended in low calcium and magnesium phosphate‐buffered saline. Male BALB/c nude mice (4–8 weeks) were injected with the ICAT‐overexpressing SW480 and control cell lines via tail vein of mice (200 μl suspension containing 2 × 10^6^ cells). Following the injection, the physiological state of the animals was observed weekly. Mice were sacrificed at 10 weeks after the injection, and the lungs were excised. After counting the tumor on the lung surface, the tumors were embedded in paraffin and sectioned.

### 2.8 Immunohistochemistry (IHC)

Tissue sections that come from animal experiments were embedded in paraffin, sectioned, and stained with hematoxylin and eosin (H&E) by Servicebio (China). Slides were dewaxed with xylene, rehydrated with graded alcohol, and incubated with 0.3% H_2_O_2_ for 10 min. The slides were placed in sodium citrate solution, heated in a microwave oven at 100°C for 5 min, then heated in medium and low heat for 25 min. The slides were sealed with goat serum for 1 h, and then incubated overnight with the following primary antibodies: anti‐ICAT (LS‐B16681, 1:500, LSBio) or anti‐Ki67 (#12202, 1:500, Cell Signaling Technology). On the following day, the slides were incubated with the respective second antibody for 1 h, then used Dako REAL EnVision Detection System (K5007) and stained with hematoxylin.

### 2.9 Immunofluorescence (IF) staining and confocal microscopy analysis

After 24 h culture, cells were transferred into a 24‐well plate with a climbing sheet at the bottom, allowing 1 × 10^4^ cells per well, and fixed using methanol for 15 min. The climbing sheet was blocked using 10% goat serum and incubated overnight at 4°C with anti‐DYKDDDDK tag (66,008‐3‐Ig, 1:50, Proteintech Group), anti‐JUP (381,616, 1:50, *ZEN‐*Bioscience). The samples were incubated with Alexa Fluor Plus 594 or 488 rabbit or mouse secondary antibodies (Life Technologies) for 1 h at room temperature. The climbing sheets were observed using a laser scanning confocal microscope (Leica TCS‐SP8).

### 2.10 Co‐immunoprecipitation mass spectrometry analysis (Co‐IP/MS) and silver staining

ICAT‐overexpressing and control cell lines were transferred to a 100‐mm Petri dish with 2 × 10^6^ cells. IP lysis buffer (Beyotime) was used for cell lysis when the cells reached confluence. Samples were placed on ice for 20 min until flocculent turbidity appeared. The samples were then centrifuged at 16,900 *g* for 10 min, and the supernatant was carefully removed and divided into two parts: the input and the remaining supernatant that mixed with anti‐Flag affinity gel (Bimake). The samples were placed on a shaker at 4°C overnight and centrifuged at 4200 *g* for 2 min, and the supernatant was then carefully discarded. The anti‐Flag affinity gel was gently washed by IP lysis buffer three times. Subsequently, a 50 μl 1× loading buffer was added to the anti‐Flag affinity gel and vortexed at 100°C for 4 min. Protein samples were analyzed using Western blotting and silver staining. Silver staining was performed using a Fast Silver Staining Kit (Beyotime).

### 2.11 STRING database

In order to predict the ICAT‐interacting candidate proteins, protein–protein interaction (PPI) analysis of ICAT was performed using STRING database. The organism was set as Homo sapiens, and the PPI score of 0.4 was considered to be the cutoff for analysis.

### 2.12 Statistical analysis

Experiments were repeated three times, and the quantitative data are presented as the mean ± standard deviation. GraphPad Prism 8.0 (GraphPad Software) was used for data analysis. The Student's *t* test was used to compare the differences between independent groups. Statistical significance was set at *p* < 0.05.

## 3 RESULTS

### 3.1 ICAT promotes cellular migration and invasion in vitro

**FIGURE 1:**
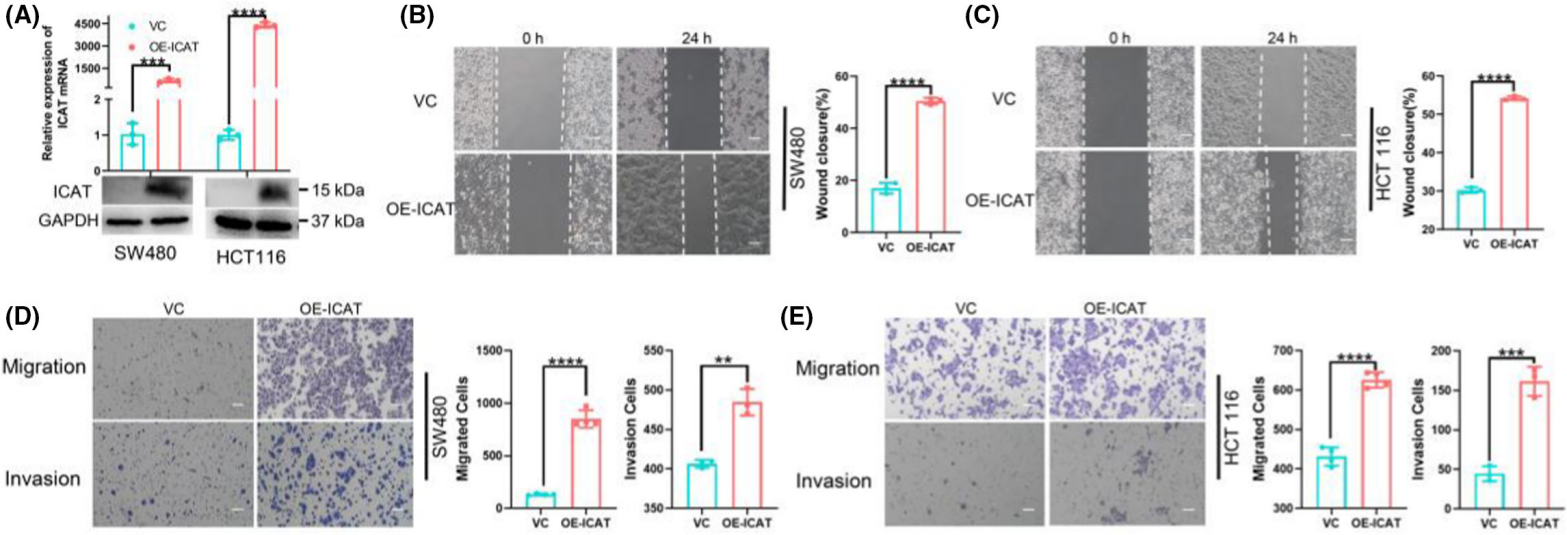
ICAT promotes cellular migration and invasion in vitro. (A) Levels of ICAT protein and mRNA expression were evaluated using Western blotting and real‐time RT‐PCR, respectively, in CRC cells overexpressing ICAT. (B and C) Wound healing assay was performed in SW480 and HCT116 cell lines overexpressing ICAT and the control cell line, respectively. Representative images (left; scale bars: 100 μm) and quantitation for the extent of wound healing (right). (D and E) A transwell assay (left; scale bars: 100 μm) shows the migration and invasion of the indicated cells. The number of migrated cells (right) groups were enumerated by ImageJ software. **p < 0.01; ***p < 0.001; ****p < 0.0001

We evaluated the effect of ICAT on mobility of CRC cells using the wound healing and transwell assays. First, the endogenous expression of ICAT in SW480 and HCT116 cells was detected (Figure S1). Then, the overexpression of ICAT of CRC cells was conducted by lentivirus infection and plasmids transfection. The efficiency of ICAT overexpression was confirmed using real‐time RT‐PCR analyses and Western blotting (Figure 1A). ICAT overexpression remarkably enhanced the migration and invasion abilities of both SW480 and HCT116 cells (Figure 1B–E).

### 3.2 ICAT promotes CRC metastasis in vivo

**FIGURE 2:**
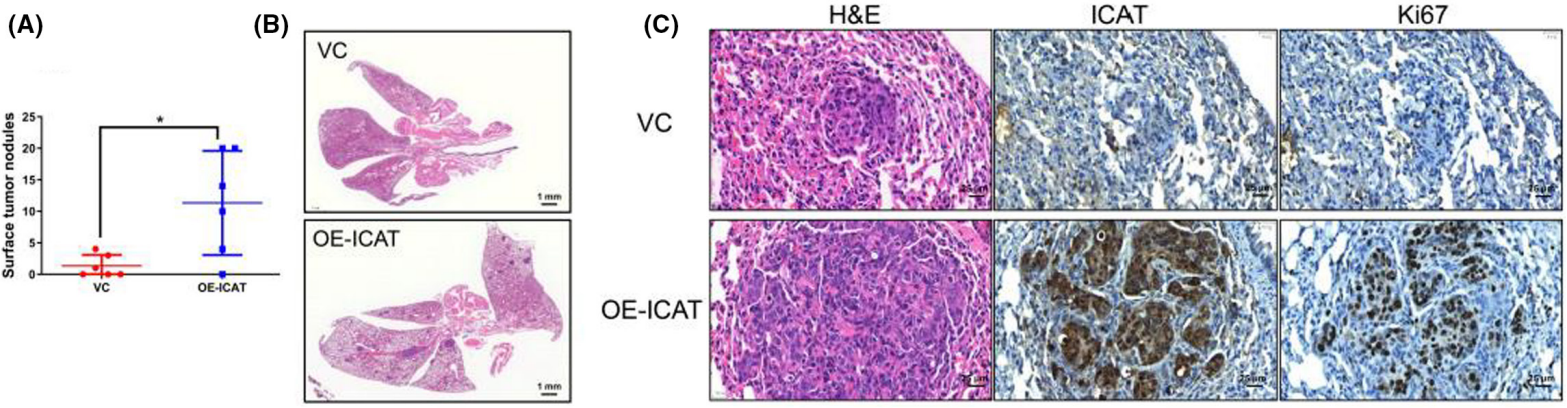
ICAT promotes CRC metastasis in vivo. (A) The ICAT‐overexpressing SW480 and control cell lines were transplanted into nude mice via tail vein injection. Counting the number of pulmonary metastases on the lung surface. (B) Representative images of H&E staining. Images were obtained using ImageViewerG. Scale bar: 1 mm. (C) Representative images of H&E staining and immunohistochemistry results for ICAT and Ki67 in lung metastatic nodules. Scale bar: 25 μm. *p < 0.05.

Because of the apparent impact of ICAT overexpression on CRC migration and invasion in vitro, we evaluated the effect of ICAT on mobility of CRC cells using a nude mouse model of lung metastasis. Consistent with the in vitro results, ICAT overexpression remarkably increased the number of lung metastatic nodules and formatted larger lung metastatic nodules (Figure 2A,B). Moreover, immunohistochemical results showed that tumor cells overexpressing ICAT exhibited high Ki67 staining. This finding suggests that ICAT‐overexpressing CRC cells have a higher proliferative ability in lung metastatic nodules (Figure 2C). Taken together, these results indicate that ICAT promotes metastasis in vivo.

### 3.3 ICAT is critical for the metastatic effects in CRC

**FIGURE 3:**
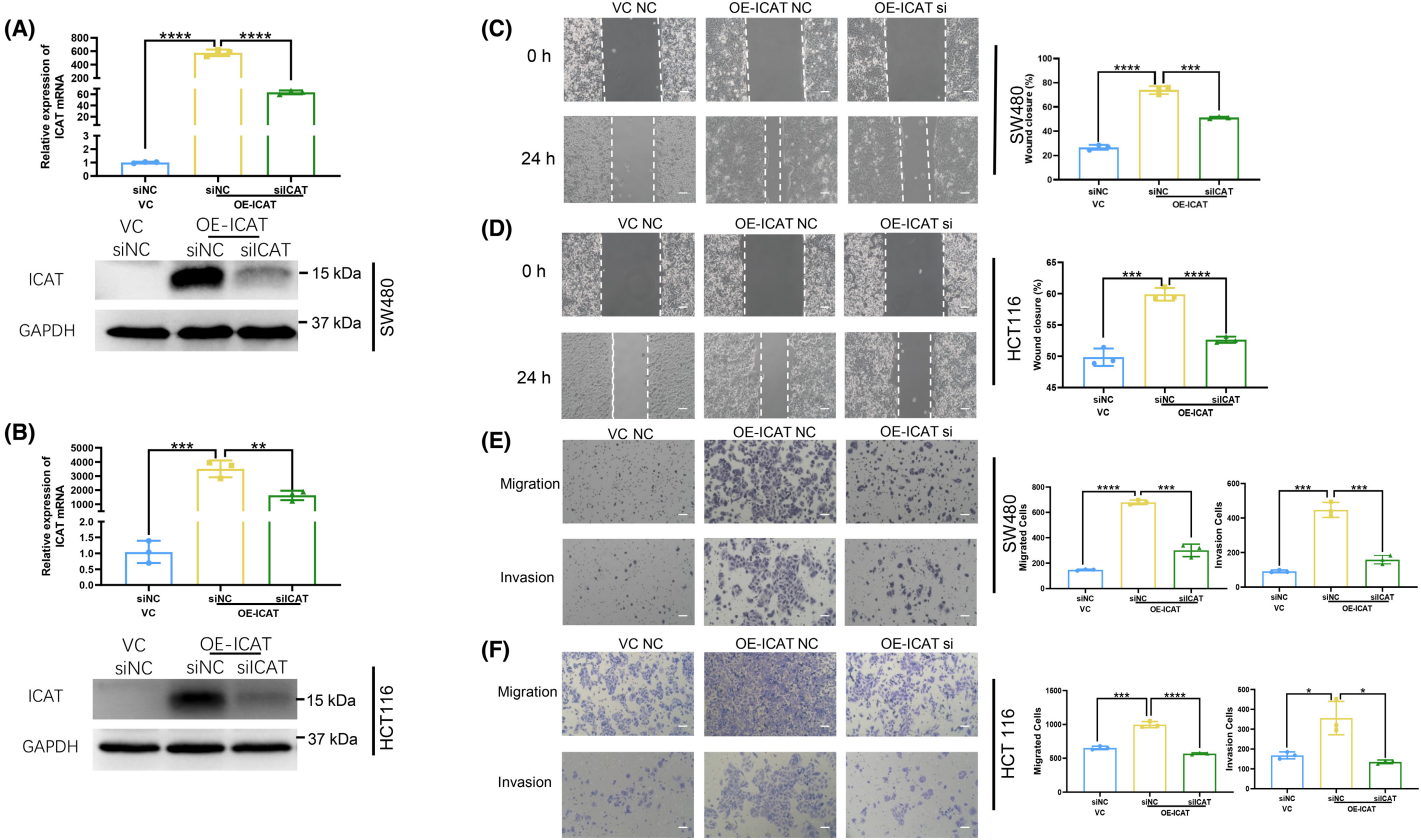
ICAT is critical for the metastatic effects in CRC. (A and B) ICAT‐overexpressing SW480 and HCT116 cell lines were transfected with siRNA against ICAT or negative control for 24 h. Western blotting and real‐time RT‐PCR analyses of ICAT expression in the transfected cells. (C and D) Wound healing assay was performed in indicated SW480 and HCT116 cells. Representative images (left; scale bars: 100 μm) and quantitation of the extent of wound healing (right). (E and F) Transwell assay (left; scale bars: 100 μm) shows the migration and invasion of the indicated cells. The number of migrated cells (right) groups were counted. *p < 0.05; **p < 0.01; ***p < 0.001; ****p < 0.0001.

To further elucidate the biological functions of ICAT, we knocked down ICAT in ICAT‐overexpressing CRC cells by transfection using an ICTA‐specific siRNA. Western blotting and real‐time RT‐PCR analyses revealed that the expression of ICAT was significantly knocked down by the siRNA (Figure 3A,B). Moreover, we verified whether ICAT knockdown could rescue ICAT‐induced migration and invasion in ICAT‐overexpressing cells. As expected, knockdown of ICAT by siRNA in ICAT‐overexpressing CRC cells significantly inhibited ICAT‐induced migration and invasion in wound healing and transwell assays (Figure 3C–F).

### 3.4 ICAT interacts with JUP in CRC cells

**FIGURE 4:**
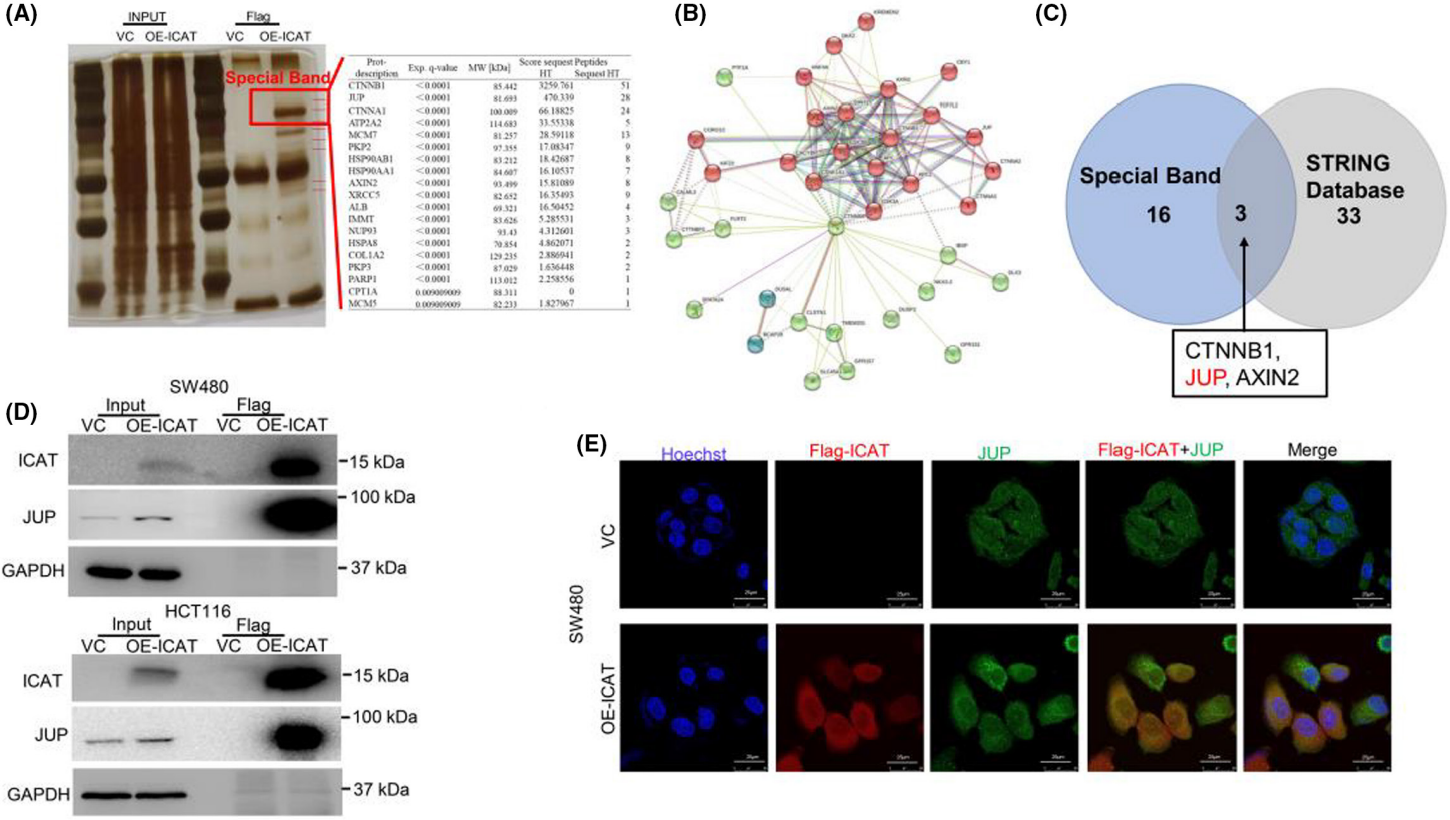
ICAT interacts with JUP in CRC cells. (A) SDS‐PAGE separation and silver staining of the immunoprecipitated proteins obtained from SW480 cells infected with lentiviruses containing ICAT‐expressing vector tagged with Flag or empty vector. The target lane was excised and analyzed using LC–MS/MS analysis. (B) Interaction network of ICAT was analyzed using the STRING database. (C) Venn diagram of candidate ICAT‐interacting proteins between protein sets of LC–MS/MS analysis and STRING database. (D) Co‐IP was used to analyze the interaction between ICAT and JUP. (E) Double IF staining for ICAT and JUP in SW480 cell line was performed to observe their location status.

To further investigate whether the ability of ICAT to promote colon cancer cell migration is related to the interaction between ICAT and other proteins, we conducted Co‐IP/MS analysis using ICAT‐overexpressing cell lines. SW480 cells infected with lentiviruses containing the ICAT‐expression vector tagged with Flag or empty vector were lysed and precipitated using anti‐Flag affinity gel. Following silver staining, a specific band was detected in the ICAT‐overexpressing cell line (Figure 4A) and subjected to LC–MS/MS analysis. We excluded peptides whose molecular weights did not match the specific band and identified candidate ICAT‐interacting peptides (Table S1). Candidate ICAT‐interacting proteins were predicted using the STRING database (Figure 4B). Several proteins were identified as components of the β‐catenin destruction complex (Figure 4C). By analyzing two sets of ICAT‐interacting candidate proteins, we focused on JUP, which had higher scores and was closely related to cell motility. Co‐IP/MS results showed a specific interaction between ICAT and JUP in both SW480 and HCT116 cells (Figure 4D). IF staining further validated that there was a considerable degree of co‐localization between ICAT and JUP in SW480 cell lines (Figure 4E).

### 3.5 JUP downregulation impaired ICAT‐induced migration and invasion in CRC cells

**FIGURE 5:**
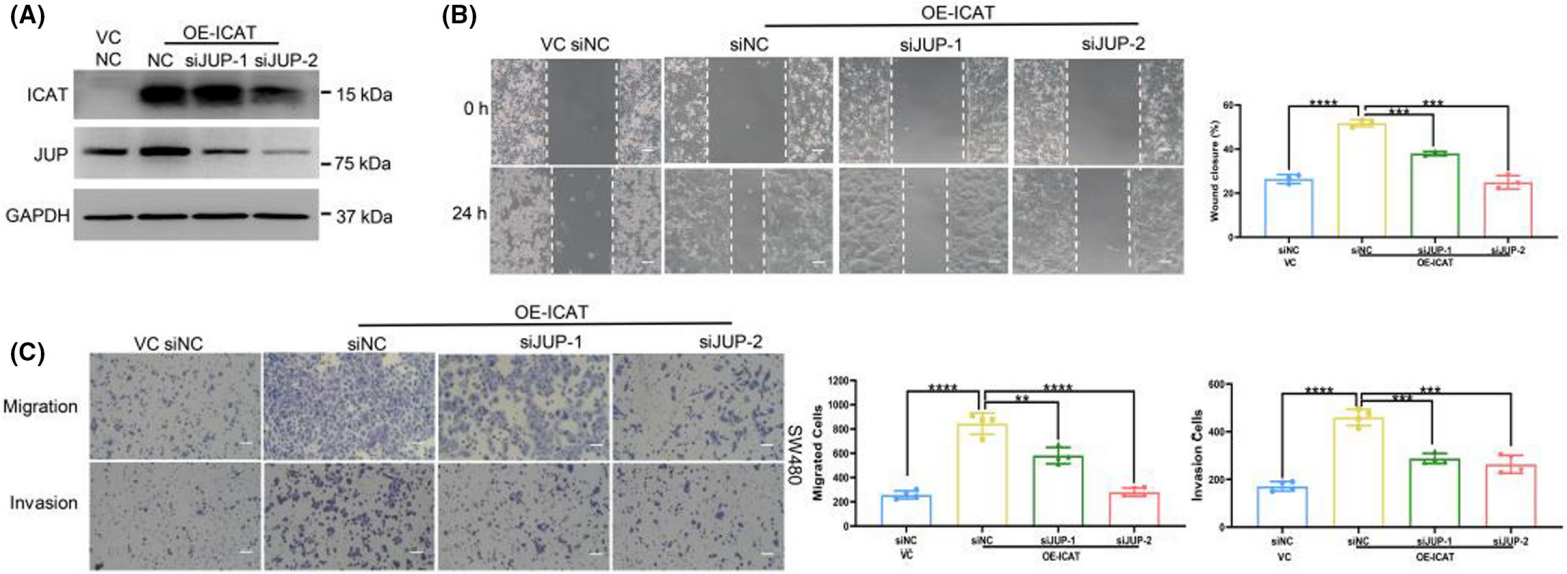
JUP downregulation impaired ICAT‐induced migration and invasion in CRC cells. (A) ICAT‐overexpressing SW480 and vector control cell lines were transfected with siRNA against either JUP or a negative control for 24 h. Western blotting was performed to analyze the expression of JUP and ICAT in transfected cells. GAPDH was used as a loading control. (B) Wound healing assay was performed in the indicated SW480 cells. Representative images (left; scale bars: 100 μm) and quantitation for the extent of wound healing (right). (C) Transwell assay (left; scale bars: 100 μm) shows the migration and invasion of the indicated cells. The number of migrated cells (right) groups were counted. **p < 0.01; ***p < 0.001; ****p < 0.0001.

To explore whether JUP plays a role in ICAT‐mediated migration and invasion in CRC cells, JUP was knocked down in ICAT‐overexpressing SW480 cells by transfection using a JUP‐specific siRNA. The knockdown efficiency of JUP was confirmed using Western blotting (Figure 5A). Next, we tested the effect of JUP knockdown on cell motility using a wound healing and transwell assays to assess cellular migration and invasion. ICAT overexpression remarkably enhanced the capacity for migration and invasion, whereas JUP knockdown reduced ICAT‐induced migration and invasion (Figure 5B,C).

### 3.6 ICAT enhanced CRC cell migration and invasion via the NF‐κB signaling pathway

**FIGURE 6:**
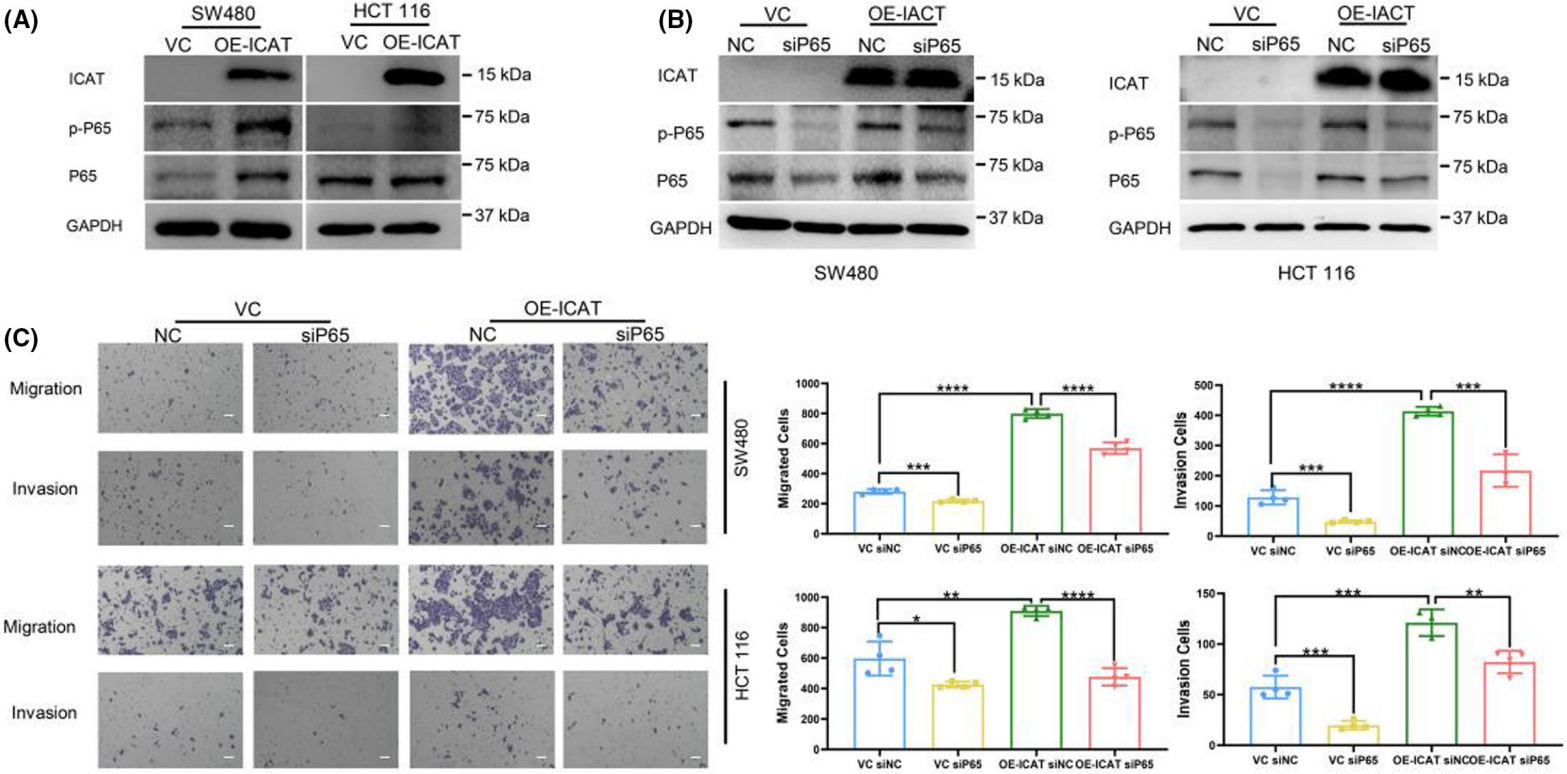
ICAT enhanced CRC cell migration and invasion via the NF‐κB signaling pathway. (A) Western blotting was used to analyze P65 and p‐P65 levels in SW480 and HCT116 ICAT‐overexpression and control cell lines, and GAPDH was used as a loading control. (B) Western blotting was used to analyze p‐P65 and P65 levels in ICAT‐overexpressing SW480 and HCT116 cell lines transfected with siRNA against P65 or negative control siRNA for 24 h, and GAPDH was used as a loading control. (C) Transwell assay (left; scale bars: 100 μm) shows the migration and invasion of the indicated cells. The number of migrated cells (right) groups were counted. *p < 0.05; **p < 0.01; ***p < 0.001; ****p < 0.0001.

The NF‐κB signaling pathway is involved in CRC metastasis.^20^, ^21^, ^22^ Hence, we hypothesized that ICAT promotes CRC cell migration and invasion by activating the NF‐κB signaling pathway. To test this hypothesis, we detected the expression of p‐P65 and P65, which represent activation of the NF‐κB pathway. We confirmed that ICAT overexpression promoted the expression of p‐P65 and P65 compared to that in the control (Figure 6A). Furthermore, we knocked down P65 using siRNA transfection and negative control (Figure 6B). We detected a change in cell mobility after P65 knockdown. Our results confirmed that P65 knockdown reduced ICAT‐induced cell invasion and migration (Figure 6C).

## 4 DISCUSSION

Our previous experiments showed that ICAT could inhibit the proliferation of CRC cells by binding to β‐catenin in the cytoplasm.^13^ However, in this study, we confirmed that ICAT could promote CRC invasion and migration, which is consistent with the premetastatic role on ICAT reported in studies of melanoma and cervical cancer.^10^, ^12^ The ICAT reflects the metastatic ability of CRC and can be developed as a potential diagnostic biomarker. Studies have shown that ICAT overexpression can block β‐catenin/APC interactions and prevent β‐catenin‐mediated APC/Axin interaction. ICAT overexpression can enhance β‐catenin stability in CRC cells containing truncated APC or stimulated by a Wnt signaling pathway stimulator. However, ICAT overexpression did not affect the stabilization of β‐catenin in CRC cells not treated with the Wnt/β‐catenin signaling pathway stimulators.^23^ Together with these findings, the function of ICAT in CRC may be environment‐specific and dynamic, and it may play different roles by interacting with other proteins. Therefore, we focused on exploring novel ICAT‐interacting proteins in this study.

Junction plakoglobin is a cell adhesion protein, and its defective expression reduces cell‐to‐cell contact and promotes migration of cancer cells in vivo.^24^ It has also been reported that JUP can induce the formation of circulating tumor cells to promote distant metastasis of breast cancer.^15^ β‐catenin is a key effector molecule in the canonical Wnt signaling pathway with strong oncogenic ability.^25^ JUP is a homolog of β‐catenin and may have the same oncogenic ability as β‐catenin. Studies have shown that JUP is a critical gene in the network that regulates the occurrence and development of CRC.^26^ The expression of JUP is enhanced in advanced CRC, and the combination of JUP and other proteins is involved in epithelial transformation and CRC liver metastasis.^27^ First, we verified the interaction between ICAT and JUP. From the perspective of protein structure, there is a possibility of an interaction between ICAT and JUP. The β‐catenin core is composed of a repeat region and serves as a platform for ICAT to bind to β‐catenin.^28^ The central repeat regions are shared features of the catenin family, while the interacting proteins bind to the central repeat domain, which may prove the validity of the interaction between ICAT and JUP.^29^ The expression of ICAT and JUP indicates the metastatic ability of CRC and provides important diagnostic value.

The NF‐κB signaling pathway is important in the tumor microenvironment and affects tumor development via various cytokines, resulting in a complex network.^30^ ICAT can bind β‐catenin and activate the NF‐κB signaling pathway, which simultaneously causes the interaction of the NF‐κB and Wnt signaling pathways. This interaction affects the proliferation and migration and improves the metastasis ability of CRC. However, further experiments are needed to determine whether ICAT, JUP, and β‐catenin can interact and have a more complex relationship or the effect of the interaction of ICAT with JUP changes the combination of ICAT and β‐catenin and the function of β‐catenin.

**FIGURE 7:**
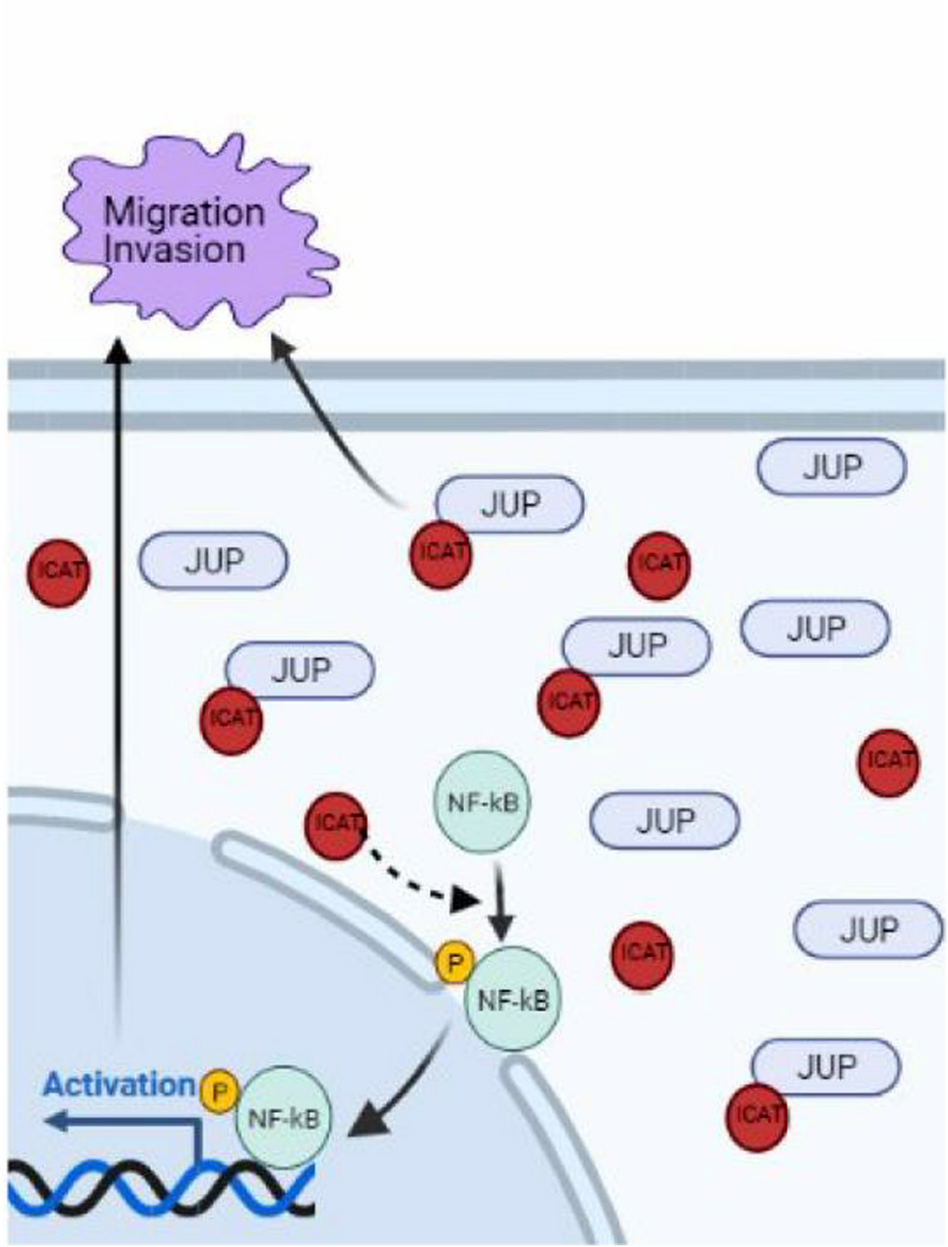
Working model of the role of ICAT in regulating CRC cells migration and invasion.

In conclusion, we propose a novel model in which ICAT promotes CRC cell migration and invasion by interacting with JUP and via the NF‐κB signaling pathway in CRC (Figure 7).

## AUTHOR CONTRIBUTIONS

All authors contributed important intellectual content during manuscript drafting and revision and approved the final draft. Zihan Wang: Investigation, Writing – Original Draft. Jiancong Hu: Writing – Original Draft, Funding acquisition. Junxiong Chen: Conceptualization, Investigation, Writing – Review & Editing. Jingdan Zhang: Investigation. Weiqian Li: Investigation. Yu Tian: Investigation. Huanliang Liu: Funding acquisition, Writing – Review & Editing. Xiangling Yang: Writing – Original Draft, Conceptualization.

## FUNDING INFORMATION

This study was supported by the National Key Clinical Discipline, Project to Attract High‐Level Foreign Experts (G20190019023), and Medical Science and Technology Foundation of Guangdong Province of China (A2019487).

## CONFLICT OF INTEREST

The authors declare no potential conflicts of interest with respect to the research, authorship, or publication of this article.

## Supporting information


Figure S1

Table S1

## Data Availability

The data used to support the findings of this study are available from the corresponding authors upon request.
