# Supplementary material for: ICAT promotes colorectal cancer metastasis via binding to JUP and activating the NF‐κB signaling pathway
Source: J Clin Lab Anal. 2022 Aug 29;36(10):e24678. doi: 10.1002/jcla.24678 (PMC9551128; doi:10.1002/jcla.24678)

**ICAT Promotes Colorectal Cancer** **Metastasis via Binding to JUP and Activating the NF-κB Signaling Pathway**

**Supplementary Information**

Supplementary Figure 1. The expression of ICAT in SW480 and HCT116.


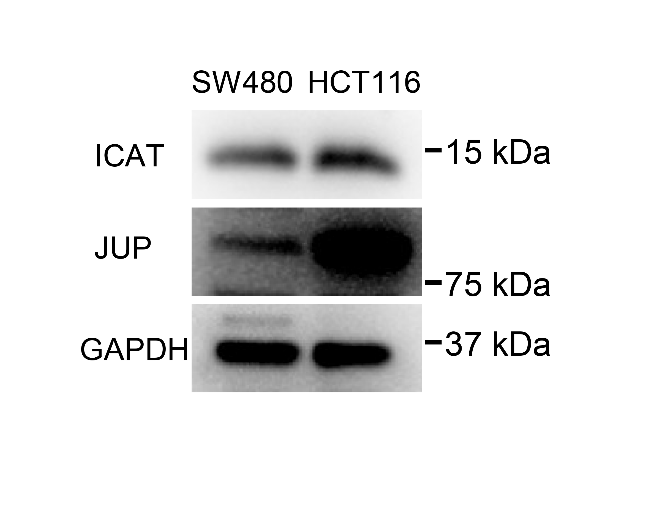

Supplement: Supplementary file 1 — Figure S1 [file JCLA-36-e24678-s001.docx]
