# Supplementary material for: ICAT promotes colorectal cancer metastasis via binding to JUP and activating the NF‐κB signaling pathway
Source: J Clin Lab Anal. 2022 Aug 29;36(10):e24678. doi: 10.1002/jcla.24678 (PMC9551128; doi:10.1002/jcla.24678)
Supplement: Supplementary file 2 — Table S1 [file JCLA-36-e24678-s002.docx]

**ICAT Promotes Colorectal Cancer** **Metastasis via Binding to JUP and Activating the NF-κB Signaling Pathway**

**Supplementary Information**

Supplementary Table 1. Raw data of candidate ICAT-interacting peptides from the LC-MS/MS analysis.

Raw data of candidate ICAT-interacting peptides from the LC-MS/MS analysis

| Prot-description | Exp. q-value | MW [kDa] | Score sequest HT | Peptides Sequest HT |
| --- | --- | --- | --- | --- |
| CTNNB1 | ＜0.0001 | 85.442 | 3259.761 | 51 |
| MYH9 | ＜0.0001 | 226.392 | 161.8951 | 50 |
| JUP | ＜0.0001 | 81.693 | 470.339 | 28 |
| CTNNA1 | ＜0.0001 | 100.009 | 66.18825 | 24 |
| KRT10 | ＜0.0001 | 58.792 | 59.63353 | 21 |
| MYH6 | ＜0.0001 | 223.596 | 50.94806 | 11 |
| KRT2 | ＜0.0001 | 65.393 | 43.52661 | 21 |
| KRT1 | ＜0.0001 | 65.999 | 41.11394 | 14 |
| ATP2A2 | ＜0.0001 | 114.683 | 33.55338 | 5 |
| MCM7 | ＜0.0001 | 81.257 | 28.59118 | 13 |
| KRT8 | ＜0.0001 | 53.671 | 21.41978 | 12 |
| KRT5 | ＜0.0001 | 62.34 | 16.71862 | 10 |
| PKP2 | ＜0.0001 | 97.355 | 17.08347 | 9 |
| HSP90AB1 | ＜0.0001 | 83.212 | 18.42687 | 8 |
| HSP90AA1 | ＜0.0001 | 84.607 | 16.10537 | 7 |
| AXIN2 | ＜0.0001 | 93.499 | 15.81089 | 8 |
| KRT18 | ＜0.0001 | 48.029 | 20.83424 | 8 |
| XRCC5 | ＜0.0001 | 82.652 | 16.35493 | 9 |
| ACTA2 | ＜0.0001 | 41.982 | 16.02813 | 4 |
| ALB | ＜0.0001 | 69.321 | 16.50452 | 4 |
| KRT13 | ＜0.0001 | 49.557 | 11.9265 | 4 |
| RPS27A | ＜0.0001 | 17.953 | 21.23706 | 4 |
| ACTB | ＜0.0001 | 41.71 | 10.32836 | 5 |
| KRT9 | ＜0.0001 | 62.027 | 6.673037 | 3 |
| MYH4 | ＜0.0001 | 222.932 | 4.54963 | 2 |
| IMMT | ＜0.0001 | 83.626 | 5.285531 | 3 |
| NUP93 | ＜0.0001 | 93.43 | 4.312601 | 3 |
| HSPA8 | ＜0.0001 | 70.854 | 4.862071 | 2 |
| ATP5F1A | ＜0.0001 | 59.714 | 5.737966 | 1 |
| COL1A2 | ＜0.0001 | 129.235 | 2.886941 | 2 |
| DCD | ＜0.0001 | 11.277 | 1.708376 | 2 |
| PKP3 | ＜0.0001 | 87.029 | 1.636448 | 2 |
| PARP1 | ＜0.0001 | 113.012 | 2.258556 | 1 |
| TUBA1B | ＜0.0001 | 50.12 | 2.421272 | 1 |
| LUC7L2 | ＜0.0001 | 46.486 | 1.758343 | 1 |
| CPT1A | 0.009009 | 88.311 | 0 | 1 |
| MCM5 | 0.009009 | 82.233 | 1.827967 | 1 |
| XRCC1 | 0.009009 | 69.434 | 2.291089 | 1 |
| LRRC14B | 0.026087 | 56.722 | 2.027807 | 1 |
| EEF1A2 | 0.026087 | 50.438 | 1.816268 | 1 |
